# Treatment at the end of life in patients with advanced melanoma. A multicenter DeCOG study of 1067 patients from the prospective skin cancer registry ADOReg

**DOI:** 10.3389/fimmu.2025.1509886

**Published:** 2025-02-24

**Authors:** Andrea Forschner, Katharina C. Kähler, Martin Gschnell, Ewan A. Langan, Carsten Weishaupt, Frank Meiss, Kai-Martin Thoms, Renate U. Wahl, Daniela Göppner, Marlene Garzarolli, Michael Sachse, Max Schlaak, Markus Reitmajer, Ivonne Kellner, Anja Gesierich, Peter Mohr, Friedegund Meier, Imke von Wasielewski, Rudolf Herbst, Jochen Utikal, Claudia Pföhler, Jens Ulrich, Patrick Terheyden, Martin Kaatz, Sebastian Haferkamp, Ulrike Leiter, Selma Ugurel, Michael Weichenthal, Carola Berking, Ralf Gutzmer, Dirk Schadendorf, Lena Nanz, Carmen Loquai

**Affiliations:** ^1^ Department of Dermatology, University Hospital, Eberhard Karls University of Tübingen, Tübingen, Germany; ^2^ Department of Dermatology, University Hospital Schleswig Holstein, Kiel, Germany; ^3^ Department of Dermatology and Allergology, University Hospital of Marburg, Philipps University Marburg, Marburg, Germany; ^4^ Department of Dermatology, University Hospital Schleswig Holstein, Lübeck, Germany; ^5^ Dermatological Sciences, University of Manchester, Manchester, United Kingdom; ^6^ Department of Dermatology, University Hospital Münster, Münster, Germany; ^7^ Department of Dermatology, Medical Center – University of Freiburg, Faculty of Medicine, University of Freiburg, Freiburg, Germany; ^8^ Department of Dermatology, University Medical Center Göttingen, Göttingen, Germany; ^9^ Department of Palliative Medicine, Medical Faculty RWTH Aachen University, Aachen, Germany; ^10^ Department of Dermatology, University Hospital of Giessen, Giessen, Germany; ^11^ Department of Dermatology, University Hospital Carl Gustav Carus, Dresden, Germany; ^12^ Department of Dermatology, Hospital of Bremerhaven, Bremerhaven, Germany; ^13^ Charité-Universitätsmedizin Berlin, Corporate member of Freie Universität Berlin and Humboldt-Universität zu Berlin, Department of Dermatology, Venereology and Allergology, Berlin, Germany; ^14^ Department of Dermatology, Helios Klinikum Erfurt, Erfurt, Germany; ^15^ Department of Dermatology, University Hospital Würzburg, Würzburg, Germany; ^16^ Department of Dermatology, Elbe Klinikum Buxtehude, Buxtehude, Germany; ^17^ Skin Cancer Center Hannover, Department of Dermatology and Allergy, Hannover Medical School, Hannover, Germany; ^18^ Skin Cancer Unit, German Cancer Research Center (DKFZ), Heidelberg, Germany; ^19^ Department of Dermatology, Venereology and Allergology, University Medical Center Mannheim, Ruprecht-Karl University of Heidelberg, Mannheim, Germany; ^20^ DKFZ Hector Cancer Institute at the University Medical Center Mannheim, Mannheim, Germany; ^21^ Department of Dermatology, Saarland University Hospital, Homburg/Saar, Germany; ^22^ Department of Dermatology, Skin Cancer Center Harz Clinics, Quedlinburg, Germany; ^23^ Department of Dermatology, SRH Wald-Klinikum Gera, Gera, Germany; ^24^ Department of Dermatology, University Hospital Regensburg, Regensburg, Germany; ^25^ Essen University Hospital, West German Cancer Center, University of Duisburg-Essen and the German Cancer Consortium (DKTK), partner site Essen/Düsseldorf, Essen, Germany; ^26^ Department of Dermatology, Uniklinikum Erlangen, CCC Erlangen-EMN, Friedrich-Alexander University Erlangen-Nürnberg (FAU), Erlangen, Germany; ^27^ Department of Dermatology, Muehlenkreiskliniken Minden and Ruhr University Bochum, Minden, Germany; ^28^ Department of Dermatology, Gesundheit Nord Klinikverbund Bremen, Bremen, Germany

**Keywords:** immune checkpoint inhibitors, melanoma, ipilimumab, nivolumab, BRAF and MEK inhibitors, end of life

## Abstract

**Background:**

Although systemic therapies have improved considerably over the last decade, up to 50% of patients with metastatic melanoma still die due to disease progression. Oncological treatment at the end-of-life phase is challenging. The aim of this study was to investigate the frequency and type of systemic therapy received by melanoma patients in their end-of-life phase.

**Methods:**

Patients with metastatic melanoma who had died between January 1, 2018 and October 31, 2022 were identified from the prospective multicenter skin cancer registry ADOReg. Study endpoints were percentage of patients who had been treated with systemic therapy within the last three months of life, timepoint of initiation of the last-line therapy, overall survival, treatment benefit and the incidence of treatment-related adverse events.

**Results:**

In total, 1067 patients from 46 skin cancer centers were included. Most of the patients (63%) had received immune checkpoint inhibitors (ICI) as last-line therapy, 22% targeted therapies (TT) and 12% chemotherapy (CTX). Comparing last-line ICI and TT, patients with TT were significantly more likely to benefit from treatment and had significantly fewer and milder treatment-related AE than patients with ICI. Even though two thirds of patients had received ICI as a last-line therapy, the majority of these patients (61%) had stopped therapy within the last 30 days of life, whereas the majority of patients with TT (66%) still continued their treatment to the end of life. We found markedly fewer patients with initiation of ICI within 30 days before their death (19%) compared to a historic cohort including patients who died in 2016 or 2017 (39%).

**Conclusion:**

Treatment approaches near the end of life have markedly changed in skin cancer centers in Germany over recent years, with ICI prescribed less frequently in the end-of-life phase. In contrast, TT are frequently administered, even within the last 30 days of life. It should also be considered that discontinuation of TT can result in rapid tumor progression. Due to the oral administration and a low rate of severe toxicity, TT appear to be a suitable treatment option, even in the end-of-life situation of melanoma patients.

## Introduction

The introduction of immune checkpoint inhibitors (ICI) and targeted therapies (TT) has drastically improved treatment options and survival rates in advanced melanoma (1–3). However, these therapies can cause serious adverse events (AE)and their use as last-line treatment has not been evaluated in prospective trials, highlighting the need for careful risk-benefit assessment of their use in the end of life. There are only a few publications on this topic and benefit assessment is rarely reported (4–7).

In 2021, the Supportive Care Committee of the Dermatologic Cooperative Oncology Group (DeCOG) published a study evaluating systemic therapies in 193 patients with advanced melanoma from 4 skin cancer centers who had died in 2016 or 2017 and were still receiving oncological systemic therapy within the last 3 months of their life (8). Most of the patients (57%) had received ICI, about one third of these patients developed severe irAE and only a small proportion (15%) benefited from last-line ICI. TT as last-line treatment resulted in a significant higher proportion of patients with benefit and fewer severe AE. Here, we assessed current trends in end of life treatment based on data from a nationwide prospective registry of patients with metastatic melanoma in Germany (ADOReg).

## Methods

Patients with metastatic melanoma who died between January 1, 2018 and October 31, 2022 were identified from the prospective multicenter skin cancer registry ADOReg of the German Dermatologic Cooperative Oncology Group (DeCOG). The ADOReg was approved by the Medical Ethics Committee of the University Duisburg-Essen (14-5921-BO), and written informed consent for participation was obtained from all patients. Study endpoints were the percentage of patients who had been on systemic therapy within the last three months of life, timepoint of initiation of the last-line therapy, overall survival, treatment benefit and the incidence of treatment-related adverse events. Furthermore, the total number of systemic non-adjuvant therapies was assessed. Benefit of last-line therapy was assessed according to the best response as documented in ADOReg. If the documented response was stable disease, mixed, complete or partial response, the patients were judged to have benefited for the treatment. Patients with progressive disease were classified as not benefiting. Patients with no available response evaluation were classified as “unknown”. Toxicity was classified according to the classification of Common Terminology Criteria for Adverse Event (CTCAE) version 5. The baseline performance status was classified according to the Eastern Cooperative Oncology Group (ECOG) performance status scale and refers to the time of initiation of last-line therapy. ECOG 0 describes patients who are fully active without restriction, ECOG 4 stands for completely disabled patients totally confined to bed. The two most common last-line systemic therapies, ICI and TT, were statistically tested for potential significant differences using chi-squared tests. When the expected cell frequency of at least one cell was less than five, Fisher’s exact test was used. Medians were compared with the median test. Overall survival (OS) was calculated as time from start of last-line systemic therapy until death. Kaplan-Meier estimates were used for OS calculation, differences between groups were assessed by two-sided log-rank tests. P values <0.05 were considered statistically significant. Statistical analyses were performed with IBM SPSS Statistics V.28. Survival curves were made with STATA/IC version 15.1.

## Results

### Patient cohort

**Figure 1:**
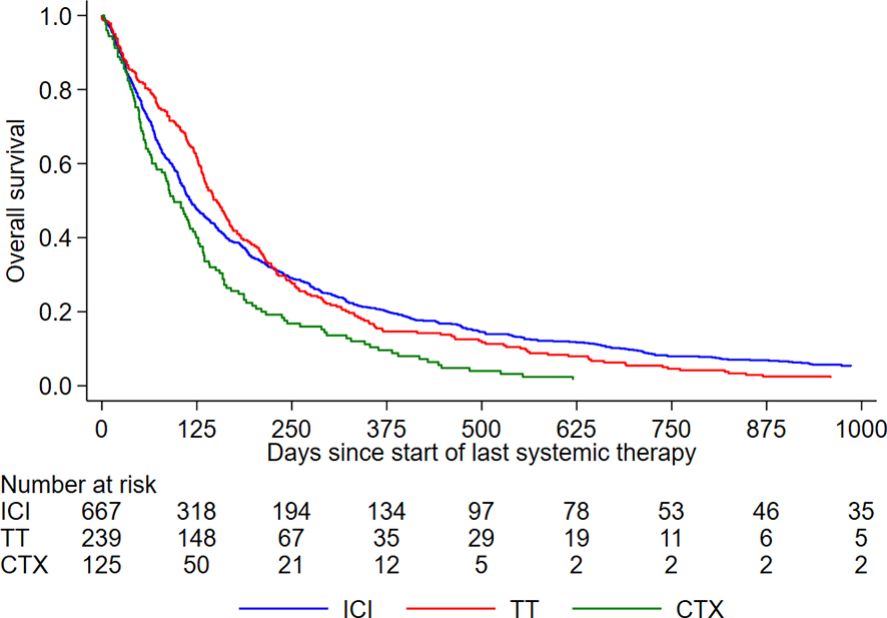
Overall survival since treatment initiation of last-line systemic therapy. ICI, immune checkpoint inhibitors; TT, Targeted therapies; CTX, Chemotherapy.

In total, 1067 patients were identified from 46 different skin cancer centers. The majority (63%) had received ICI as last-line therapy, 22% had received TT and 12% chemotherapy (CTX). More than half of all patients with ICI (52%) had combined CTLA-4 and PD-1 antibodies and most patients with targeted therapy (89%) had a combination of BRAF- and MEK inhibitors (**Table 1**). About three quarters of the patients (74%) underwent systemic therapy within 90 days of death, approximately half of the patients within 30 days of death. The last line of therapy had been started within the last 30 days before death in 13% of patients; within 7 days before death in 2%. The median time from treatment initiation to death was 127 days, interquartile range (IQR) 57 – 279 days. Almost one third of the patients had brain metastases and in half of the patients lactate dehydrogenase (LDH) was elevated baseline to last treatment initiation. In one third, baseline performance status according to the Eastern Cooperative Oncology Group (ECOG) was ≥1, in 40% of the patients ECOG was unknown. Approximately 40% of the patients had received three or more systemic therapies before death. This percentage was highest in patients with CTX as the last-line treatment (69%), followed by TT (50%) and ICI (29%). Among all patients with BRAF V600 mutation, 52% had TT as last-line therapy. About 44% of the patients with last-line TT had already been treated with TT at an earlier time point, i.e. they received TT as a re-challenge. The proportion of patients with benefit from last-line therapy was highest in patients with TT, followed by ICI and CTX (**Supplementary Table S1**). OS was significantly worse for patients with CTX as the last-line therapy (p=0.004) (**Figure 1**).

**Table 1:** Patient characteristics (n=1067).

	Median	IQR
Age at death (years)	68	57-78
Days between start of last systemic therapy and death	127	57-279
Days between end of last systemic therapy and death	35	12-97
	No. patients	%
Sex		
Female	391	36.6
Male	676	63.4
Melanoma type		
Cutaneous	719	67.4
Acral	70	6.6
Unknown primary	133	12.5
Mucosal	48	4.5
Ocular	33	3.1
Not further specified	64	6.0
BRAF mutation		
Present	462	43.3
Absent	478	44.8
Unknown	127	11.9
Number of systemic therapies until death		
1	357	33.5
2	293	27.5
≥3	417	39.1
Ninety days before death under systemic therapy		
Yes	785	73.6
No	282	26.4
Thirty days before death under systemic therapy		
Yes	482	45.2
No	585	54.8
Start systemic therapy within ninety days before death		
Yes	404	37.9
No	663	62.1
Start systemic therapy within thirty days before death		
Yes	138	12.9
No	929	87.1
Type of last systemic therapy		
Immune checkpoint inhibitor PD-1 antibody + CTLA-4 antibody n= 349 PD-1 antibody n=281 CTLA-4 antibody n=36 Not specified n=1	667	62.5
Targeted therapy	239	22.4
BRAF inhibitor + MEK inhibitor n=213 MEK inhibitor n=13 BRAF inhibitor n=8 Other n=5		
Chemotherapy	125	11.7
Combined targeted therapy or chemotherapy with immune checkpoint inhibitor	11	1.0
Other	25	2.3
ECOG at start of last systemic therapy		
0	316	29.6
1	225	21.1
≥2	102	9.6
Unknown	424	39.7
Brain metastasis at start of last systemic therapy		
Present	331	31.0
Absent	736	69.0
LDH at start of last systemic therapy		
Normal	299	28.0
1-fold elevated	335	31.4
≥2-fold elevated	193	18.1
Unknown	240	22.5

### Comparison between ICI and TT

Even though the majority (63%) of patients had received ICI as last-line therapy within their last 3 months of life, almost two thirds (61%) of these patients with ICI stopped therapy within the last 30 days of life. In contrast, the majority of patients (66%) with TT still continued TT within the last days of life (p<0.001) (**Table 2**). The percentage of patients with treatment initiation within the last 30 days of life was similar in both groups, i.e., 13% of patients with ICI and 12% of patients with TT.

**Table 2:** Immune checkpoint inhibitor versus targeted therapy as last-line treatment.

	Immune checkpoint inhibitor (n=667)	Targeted therapy (n=239)	P-value
Median age at start of last systemic therapy (IQR)	70 (60–78)	64 (52-75)	<0.001
Median time between start of last systemic therapy and death (IQR)	117 (54-297)	151 (75-270)	<0.001
Median time between end of last systemic therapy and death (IQR)	44 (18-120)	15 (1-50)	<0.001
**Number of systemic therapies until death**			<0.001
1	284 (42.6)	61 (25.5)	
2	190 (28.5)	60 (25.1)	
≥3	193 (28.9)	118 (49.4)	
**Ninety days before death under systemic therapy**			<0.001
Yes	464 (69.6)	204 (85.4)	
No	203 (30.4)	35 (14.6)	
**Thirty days before death under systemic therapy**			<0.001
Yes	259 (38.8)	158 (66.1)	
No	408 (61.2)	81 (33.9)	
**Start systemic therapy within ninety days before death**			0.002
Yes	266 (39.9)	68 (28.5)	
No	401 (60.1)	171 (71.5)	
**Start systemic therapy within thirty days before death**			0.675
Yes	88 (13.2)	29 (12.1)	
No	579 (86.8)	210 (87.9)	
**ECOG at start of last systemic therapy**			0.043
0	214 (32.1)	56 (23.4)	
1	139 (20.8)	59 (24.7)	
≥2	52 (7.8)	27 (11.3)	
Unknown	262 (39.3)	97 (40.6)	
**Brain metastasis at start of last systemic therapy**			0.057
Present	193 (28.9)	85 (35.6)	
Absent	474 (71.1)	154 (64.4)	
**LDH at start of last systemic therapy**			<0.001
Normal	220 (33.0)	49 (20.5)	
1-fold elevated	195 (29.2)	83 (34.7)	
≥2-fold elevated	115 (17.2)	38 (15.9)	
Unknown	137 (20.5)	69 (28.9)	
**Benefit of last systemic therapy**			0.001
Yes	167 (25.0)	89 (37.2)	
No	298 (44.7)	85 (35.6)	
Unknown	202 (30.3)	65 (27.2)	
**Toxicity of last systemic therapy**			0.002
Yes	220 (33.0)	53 (22.2)	
No	447 (67.0)	186 (77.8)	
Maximal grade			Fisher’s exact test: 0.202
1-2	115 (17.2)	33 (13.8)	
3-4	94 (14.1)	16 (6.7)	
5	3 (0.4)	2 (0.8)	
Unknown	8 (1.2)	2 (0.8)	

**Figure 2:**
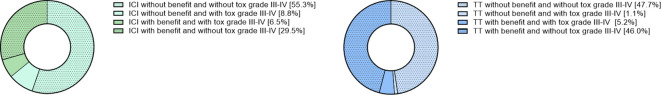
Treatment benefit and toxicity of last-line ICI (green, left part) and TT (blue, right part).

Considering the number of systemic therapies that had been applied before death, patients with TT had had significant more treatment lines, half of them ≥ 3. Patients with ICI had received in more than 40% of the cases only one systemic therapy before death (p<0.001). LDH baseline was normal in one third of patients with ICI and in 21% of patients with TT (p<0.001). The proportion of patients who benefited from last-line treatment was significantly higher in the TT group (37%) compared to the ICI group (25%). Though, it has to be considered that the benefit had not been documented in about one third of patients of both groups. When only patients with known benefit status are considered, the difference was even greater: 51% of patients with TT benefited from last-line treatment compared to 36% of patients with ICI. Regarding treatment-related AEs, 33% of patients with ICI and 22% of patients with TT had toxicity due to last-line therapy, respectively (**Figure 2**; **Table 3**). The percentage of CTCAE toxicity grade 3 or 4 was double for ICI (14%) compared to the TT group (7%). Regarding OS since treatment initiation of last-line therapy, there was no significant difference between the ICI and TT group (p=0.791) (**Figure 1**).

**Table 3:** Treatment-related adverse events with immune checkpoint inhibitor and targeted therapy as last-line treatment.

	Immune checkpoint inhibitor (n=667)	Targeted therapy (n=239)
Colitis	84 (12.6)	9 (3.8)
Endocrinologic AE	40 (6.0)	0 (0.0)
Skin toxicity	40 (6.0)	9 (3.8)
Hepatobiliary/pancreatic AE	37 (5.5)	3 (1.3)
Fatigue/anorexia	31 (4.6)	11 (4.6)
Lung toxicity	25 (3.7)	7 (2.9)
Neurological/musculoskeletal AE	17 (2.5)	8 (3.3)
Pain/arthralgia	15 (2.2)	5 (2.1)
Fever	9 (1.3)	12 (5.0)
Hematological AE	9 (1.3)	2 (0.8)
Nephrological AE	9 (1.3)	1 (0.4)
Other	51 (7.6)	19 (7.9)

## Discussion

Patients with TT were not only significantly more likely to benefit from therapy, they also suffered significantly fewer treatment-related AE compared to patients who received ICI as the last line therapy. These results confirm the data from our previously published study that included a notably smaller cohort (6). At that time, it was assumed that the large number of patients that had commenced the newly approved ICI therapy may have reflected unrealistic expectations of treatment response (8). In the cohort of patients who had died in 2016-2017, 85% of the patients with last-line ICI had ECOG performance status ≥1. In the present study only 47% of patients with known performance status had ECOG ≥1. Similarly, the proportion of patients with ICI and normal LDH values was significantly lower (26%) in the historical cohort compared to the current cohort (42%). In both cohorts, the ECOG and LDH values of the TT group were significantly worse compared to the respective ICI cohort and the proportion of patients who benefited from last-line TT was significantly higher. This further supports the approach of re-challenge with BRAF and MEK inhibitors in melanoma (9). It should also be mentioned at this point, that TT are an oral medication that can be administered by patients at home, whereas ICI have to be administered intravenously at medical centers. In case of toxicity, treatment-related AE usually cease with treatment discontinuation and hospitalization is only required in rare cases. It should also be considered that if TT are discontinued because of disease progression, even faster metastatic growth is commonly observed. Due to the oral administration and a low rate of high-grade toxicity, TT appear to be a suitable treatment option, even at the end of life. In view to the limited prognosis in patients with re-challenge of TT this is an important aspect to be considered. Patients with CTX had worst survival and lowest benefit rate. These results confirm the limited efficacy of CTX in patients with advanced melanoma (10). Considering patients’ wishes regarding end of life situation, most of them wish to die at home. Therefore, physicians should honestly discuss potential benefit and expected effort/toxicity of treatments near end of life. CTX is in most cases associated with hospitalization and significant toxicity, so there is a high risk that CTX will do more harm than good to patients at the end of life (11–13).

We found a significantly lower percentage of patients for whom ICI was initiated within 30 days of death (19%) compared to the historic cohort (39%). It is evident that a learning process has taken place here, which has probably led to a more realistic assessment of risk and benefit at the end of life. This is also supported by survival analyses. In the historic cohort, OS since treatment initiation of last-line therapy was significantly worse in patients with last-line ICI compared to TT. In the present study, which reflects the approach of the centers in the period well after approval of ICI, there is no difference, which is probably due to the fact that ICI treatment was no longer initiated in patients approaching the end of life.

In a retrospective cohort study of a US national clinical database of patients with metastatic melanoma and other cancer types between 2016 and 2019, it was observed that the number of patients with initiation of ICI within 1 months before death increased much more than other therapies decreased, thus, ICI were added as an additional therapy at the end of life. The authors concluded that there was an unrealistic hope in ICI even within the last days of life. They found that academic centers and centers with high numbers of patients were more reluctant to initiate ICI within 30 days before death. The authors speculated that these centers might manage severe irAE more often and prescribe ICI with greater caution to “borderline” candidates, i.e., with reduced ECOG near end of life (6).

Furthermore, it is well known that the availability of new therapies is usually accompanied by a simultaneous increase in their prescription. However, the transfer of study data to a real-world setting is challenging and should not simply be adopted without reflection. With regard to ICI, this phenomenon was particularly pronounced, perhaps because patients and physicians tended to underestimate potential toxicities and overestimate potential benefits of ICI. It has been shown that the use of anti-cancer therapies near end of life in patients with advanced melanoma who died between 2013 and 2017 has increased significantly since ICI had been approved (14).

In the present evaluation of a large real-world cohort we found that the percentage of patients with treatment initiation within the last 30 days of death has significantly decreased over time, which may reflect an improved ability of physicians to assess prognosis more realistically and to consider potential risks of ICI more carefully. Treatment approaches near end of life have apparently changed over recent years in dermato-oncology centers in Germany, which can certainly be attributed to an increase in knowledge in the care for patients with advanced melanoma at the end of their lives.

A more realistic assessment of available treatment options and open and honest conversations with patients and their families will enable them to plan the last phase of their lives according to their wishes and needs at the end of life (15, 16). It is important to recognize that there is a fine line between providing effective treatments that have a positive impact on patients’ lives and overtreatment causing more harm than good. Early integration of palliative care rather than aggressive systemic therapy has been shown to improve quality of life and even prolong survival, as has been shown in lung cancer (17). A recent publication addressed factors that contribute to overtreatment of cancer patients at the end of life. The authors encourage open, unbiased conversations, early implementation of palliative care and considering patient’s individual goals in order to avoid overtreatment as far as possible (18).

There are some limitations of our study. First, the information on benefit was not always documented in the ADOReg registry. The proportion of patients with missing data on benefit was about one third in all treatment categories (ICI, TT and CTX). When calculating the percentage of patients with benefit, we only included patients with known data. The lack of response assessment is likely due to a deteriorating performance status of this advanced cohort and the increasing inability to undergo imaging procedures. The percentage of patients with toxicity in general and specifically of grade 3 or 4 was low. This might be due to insufficient documentation in the medical files, which are reviewed by the documentaries of the skin cancer centers. However, there is no reason to suggest this biased the results in terms of TT or IT as it applies to all treatment types and should not detract from the conclusion that ICI patients had more often and more severe toxicity compared to TT. The strength of this study is the high number of patients and the accurate documentation of data on treatment initiation, death and patients’ treatments before death. The results of our data are important because they reflect the real-world situation of melanoma patients near end of life in more than 40 different skin cancer centers in a very large cohort. National registries such as the ADOReg are extremely important to obtain such data.

We believe that our study makes a significant contribution to the care of patients with metastatic melanoma at the end of life. Treatment approaches have obviously changed over years in Germany, with a decrease of the use of ICI at the end of life. In contrast, TT are still of high relevance, even within the last 30 days of life. Due to the oral administration and a low rate of high-grade toxicity, TT appear to be a suitable palliative treatment option, even at the end of life.

## Data Availability

The raw data supporting the conclusions of this article will be made available by the authors, without undue reservation.
