# Supplementary material for: Treatment at the end of life in patients with advanced melanoma. A multicenter DeCOG study of 1067 patients from the prospective skin cancer registry ADOReg
Source: Front Immunol. 2025 Feb 24;16:1509886. doi: 10.3389/fimmu.2025.1509886 (PMC11891187; doi:10.3389/fimmu.2025.1509886)
Supplement: Supplementary file 1 [file Table1.docx]

**Supplementary Table 1** **Immune checkpoint inhibitor, targeted therapy, chemotherapy as last-line treatment**

|  | **Immune checkpoint inhibitor (n=667)** | **Targeted therapy (n=239)** | **Chemotherapy (n=125)** |
| --- | --- | --- | --- |
| Median age at start of last systemic therapy (IQR) | 70 (60-78) | 64 (52-75) | 65 (56-74) |
| Median time between start of last systemic therapy and death (IQR) | 117 (54-297) | 151 (75-270) | 95 (46-184) |
| Median time between end of last systemic therapy and death (IQR) | 44 (18-120) | 15 (1-50) | 40 (16-83) |
| **Number of systemic therapies until death** |  |  |  |
| 1 | 284 (42.6) | 61 (25.5) | 4 (3.2) |
| 2 | 190 (28.5) | 60 (25.1) | 35 (28.0) |
| ≥3 | 193 (28.9) | 118 (49.4) | 86 (68.8) |
| **Ninety days before death under systemic therapy** |  |  |  |
| Yes | 464 (69.6) | 204 (85.4) | 98 (78.4) |
| No | 203 (30.4) | 35 (14.6) | 27 (21.6) |
| **Thirty days before death under systemic therapy** |  |  |  |
| Yes | 259 (38.8) | 158 (66.1) | 54 (43.2) |
| No | 408 (61.2) | 81 (33.9) | 71 (56.8) |
| **Start systemic therapy within ninety days before death** |  |  |  |
| Yes | 266 (39.9) | 68 (28.5) | 61 (48.8) |
| No | 401 (60.1) | 171 (71.5) | 64 (51.2) |
| **Start systemic therapy within thirty days before death** |  |  |  |
| Yes | 88 (13.2) | 29 (12.1) | 18 (14.4) |
| No | 579 (86.8) | 210 (87.9) | 107 (85.6) |
| **ECOG at start of last systemic therapy** |  |  |  |
| 0 | 214 (32.1) | 56 (23.4) | 36 (28.8) |
| 1 | 139 (20.8) | 59 (24.7) | 21 (16.8) |
| ≥2 | 52 (7.8) | 27 (11.3) | 21 (16.8) |
| Unknown | 262 (39.3) | 97 (40.6) | 47 (37.6) |
| **Brain metastasis at start of last systemic therapy** |  |  |  |
| Present | 193 (28.9) | 85 (35.6) | 48 (38.4) |
| Absent | 474 (71.1) | 154 (64.4) | 77 (61.6) |
| **LDH at start of last systemic therapy** |  |  |  |
| Normal | 220 (33.0) | 49 (20.5) | 17 (13.6) |
| 1-fold elevated | 195 (29.2) | 83 (34.7) | 49 (39.2) |
| ≥2-fold elevated | 115 (17.2) | 38 (15.9) | 34 (27.2) |
| Unknown | 137 (20.5) | 69 (28.9) | 25 (20.0) |
| **Benefit of last systemic therapy** |  |  |  |
| Yes | 167 (25.0)  *[20 CR, 55 PR, 74 SD, 18 MR]* | 89 (37.2)  *[5 CR, 36 PR, 30 SD, 18 MR]* | 25 (20.0)  *[1 CR, 5 PR, 14 SD, 5 MR]* |
| No | 298 (44.7) | 85 (35.6) | 54 (43.2) |
| Unknown | 202 (30.3) | 65 (27.2) | 46 (36.8) |
| **Toxicity of last systemic therapy** |  |  |  |
| Yes | 220 (33.0) | 53 (22.2) | 27 (21.6) |
| No | 447 (67.0) | 186 (77.8) | 98 (78.4) |
| Maximal grade |  |  |  |
| 1-2 | 115 (17.2) | 33 (13.8) | 17 (13.6) |
| 3-4 | 94 (14.1) | 16 (6.7) | 8 (6.4) |
| 5 | 3 (0.4) | 2 (0.8) | 1 (0.8) |
| Unknown | 8 (1.2) | 2 (0.8) | 1 (0.8) |

CR: complete remission, PR: partial remission, SD: stable disease, MR: mixed response
